# Molt-dependent transcriptomic analysis of cement proteins in the barnacle *Amphibalanus amphitrite*

**DOI:** 10.1186/s12864-015-2076-1

**Published:** 2015-10-24

**Authors:** Zheng Wang, Dagmar H. Leary, Jinny Liu, Robert E. Settlage, Kenan P. Fears, Stella H. North, Anahita Mostaghim, Tara Essock-Burns, Sarah E. Haynes, Kathryn J. Wahl, Christopher M. Spillmann

**Affiliations:** Center for Bio/Molecular Science and Engineering, Naval Research Laboratory, Washington, DC 20375 USA; Virginia Bioinformatics Institute, 1015 Life Science Circle, Blacksburg, VA 24061 USA; Chemistry Division, Naval Research Laboratory, Washington, DC 20375 USA; Present address: Eastern Virginia Medical School, 700 West Olney Road, Norfolk, VA 23507 USA; Present address: Duke University Marine Laboratory, 135 Duke Marine Lab Rd. Beaufort, North Carolina, 28516 USA; Present address: Department of Chemistry, University of Michigan, 930 N. University Avenue, Ann Arbor, MI 48109 USA

**Keywords:** Cement protein, Molt cycle, Transcriptome, Proteome, Barnacle, *Amphibalanus amphitrite*

## Abstract

**Background:**

A complete understanding of barnacle adhesion remains elusive as the process occurs within and beneath the confines of a rigid calcified shell. Barnacle cement is mainly proteinaceous and several individual proteins have been identified in the hardened cement at the barnacle-substrate interface. Little is known about the molt- and tissue-specific expression of cement protein genes but could offer valuable insight into the complex multi-step processes of barnacle growth and adhesion.

**Methods:**

The main body and sub-mantle tissue of the barnacle *Amphibalanus amphitrite* (basionym *Balanus amphitrite*) were collected in pre- and post-molt stages. RNA-seq technology was used to analyze the transcriptome for differential gene expression at these two stages and liquid chromatography-mass spectrometry/mass spectrometry (LC-MS/MS) was used to analyze the protein content of barnacle secretions.

**Results:**

We report on the transcriptomic analysis of barnacle cement gland tissue in pre- and post-molt growth stages and proteomic investigation of barnacle secretions. While no significant difference was found in the expression of cement proteins genes at pre- and post-molting stages, expression levels were highly elevated in the sub-mantle tissue (where the cement glands are located) compared to the main barnacle body. We report the discovery of a novel 114kD cement protein, which is identified in material secreted onto various surfaces by adult barnacles and with the encoding gene highly expressed in the sub-mantle tissue. Further differential gene expression analysis of the sub-mantle tissue samples reveals a limited number of genes highly expressed in pre-molt samples with a range of functions including cuticular development, biominerialization, and proteolytic activity.

**Conclusions:**

The expression of cement protein genes appears to remain constant through the molt cycle and is largely confined to the sub-mantle tissue. Our results reveal a novel and potentially prominent protein to the mix of cement-related components in *A. amphitrite*. Despite the lack of a complete genome, sample collection allowed for extended transcriptomic analysis of pre- and post-molt barnacle samples and identified a number of highly-expressed genes. Our results highlight the complexities of this sessile marine organism as it grows via molt cycles and increases the area over which it exhibits robust adhesion to its substrate.

**Electronic supplementary material:**

The online version of this article (doi:10.1186/s12864-015-2076-1) contains supplementary material, which is available to authorized users.

## Background

Adhesion related to biological processes is often important to maintain proper function, and this is especially true of sessile marine organisms where substrate adhesion is critical for survival. A prominent example is the barnacle, which first relies on temporary adhesion on a suitable substrate as a cyprid and then transitions to permanent attachment as the animal undergoes metamorphosis to a sessile juvenile and matures to an adult with either a calcareous or membranous base attached to the underlying surface [1]. Barnacles are arthropods in the Crustacea subphylum and are partly characterized by their growth via molt cycles. Molting in adult acorn barnacles is evidenced by shedding of the exuvia as well as the formation of radial ecdysal lines underneath the animal. Much effort has been dedicated to understanding the intertwined processes of growth and adhesion as well as the composition and the molecular mechanisms that lead to a strongly adhered organism. These efforts are complicated by the presence of a rigid outer calcareous shell, under which cement components are delivered and deposited by the barnacle under the leading edge of the barnacle/substrate interface and sheltered from the external marine environment. As with growth, evidence suggests the process of adhesion occurs in a cyclic, multi-step manner synchronized with the molt cycle [2, 3]. The means by which cement precursor materials are delivered to the leading edge of the barnacle/substrate interface are not completely understood, yet a network of capillaries originating from cement glands and terminating at ducts at the substrate interface are thought to play a prominent role [2–4].

Barnacle cement itself is composed predominantly of protein [5–7], though other materials, such as lipids, polysaccharides and biomineralization components, have also been found at the barnacle-substrate interface [1]. Several putative cement proteins have been identified and studied, mainly through solubilization and analysis of cement plaques that remained on substrates following removal of the barnacle shell and body [7–12]. These include cp100k, cp68k, cp52k, cp20k, cp19k, where the number corresponds with the protein molecular weight in daltons. Detailed structural information about each protein remains unknown, though amino acid composition and conserved and/or repeat subunits have shed light on potential function [9, 12, 13]. Additionally, advances in gene sequencing technology have aided in the development of cDNA libraries as well as the discovery of genes that encode cement protein homologues in different barnacle species [14–16]. This knowledge continues to fill in the picture of how barnacles are able to create a robust adhesive interface in a relatively harsh marine environment.

With this backdrop, we set out to test the hypothesis that cement proteins are actively regulated at different stages of the molt cycle and in different tissues of adult barnacles. Molting of several adult *Amphibalanus amphitrite* (basionym *Balanus amphitrite*) barnacles was tracked based on shedding of the exuvia under controlled laboratory conditions. Protein and mRNA were collected from the sub-mantle tissue (containing the cement glands) of barnacles in pre- and post-molt stages. Material was also collected from the main body of adult barnacles for comparison. Using RNA-Seq technology, the expression levels of cement protein genes in the sub-mantle tissue of pre- and post-molt samples were found to be the same, suggesting these proteins are not regulated at the transcript level when synchronized to the molt cycle. Comparison of cement protein gene expression in the sub-mantle tissue versus the main barnacle body revealed significantly elevated levels in sub-mantle tissue. These results fit with the current understanding of barnacle anatomy, where cement pre-cursor material is thought to be produced and stored in cement glands located in the sub-mantle tissue. We also report the discovery of a novel 114kD protein, named Aacp114k, and found its transcript to be highly expressed in the sub-mantle tissue and the protein identified in barnacle cement secretions collected at the barnacle/substrate interface. This protein shares significant homology to a previously identified 100kD cement protein in *A. amphitrite* as well as others identified in the barnacle species *Megabalanus rosa* and *Teraclita japonica formosana* [7, 15, 16].

In the absence of a complete genome, the information collected from the comparison of pre- and post-molt samples allowed for a more general analysis of differential gene expression in these two stages. A number of genes were identified as being up-regulated in the sub-mantle tissue predominantly in pre-molt barnacle samples with a range of functions including cuticular development, biominerialization, and proteolytic activity. Collectively, these results add a novel and potentially prominent protein to the mix of cement-related components and further our understanding of the complex processes at play as barnacles grow via cyclic molting and extend their adhesive area to substrates.

## Results

### Sample collection and molt stage confirmation

**Fig. 1:**
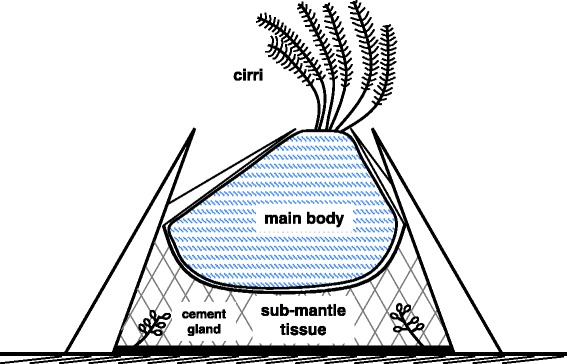
Simplified schematic of adult acorn barnacle cross section. The three sections used in this study are shown. Cirri were used to confirm the molt stage of each sample, while the main body and sub-mantle tissue were collected for protein and mRNA analysis. Note the sub-mantle tissue contains the cement glands

To test our hypothesis that cement protein genes are differentially expressed during the molt cycle of barnacles and in separate regions of the body, stable laboratory conditions were established for barnacles. Adult *A. amphitrite* individually reattached on glass slides were maintained and monitored in an incubator providing control over several variables including temperature, humidity, salinity, and light/dark cycles to minimize stressful environmental variables. Barnacles exhibiting stable, regular molting were selected in either a pre-molt or post-molt stage and the cirri and sub-mantle tissue were collected for further analysis. A barnacle schematic showing the cirri, main body, and sub-mantle tissue is shown in Fig. 1.

**Fig. 2:**
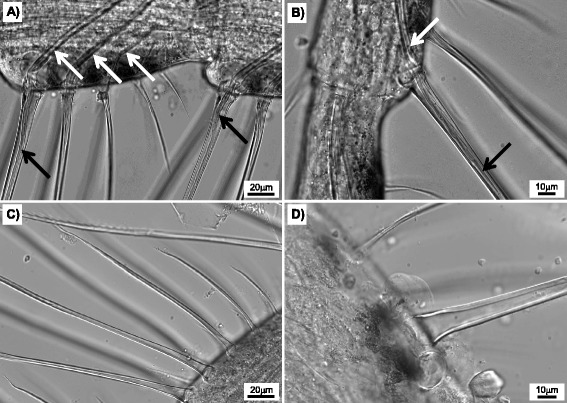
Rami from adult barnacle *A. amphitrite* highlighting the setae in pre- and post-molt stages. **a-b**) Images of several setae from barnacles collected in pre-molt (proecdysis) stage. Black arrows indicate the presence of new setae development prior to molting, and white arrows indicate invaginations of the newly developing setae. **c-d**) Images of setae from barnacles collected in post-molt stages as indicated by the absence of new setae or invaginations and an expanded setal matrix

Each barnacle used in this study had the cirri removed for microscopic determination of the molt cycle based on the analysis of Davis et al. [17]. They reported significant temporal variability of the molt stages, yet the most common stages observed in the current study were the pre-molt, proecdysis (stage D) and the post- and interecdysis stages (B and C). Postecdysis stage A is a very short period of ~4 h immediately following a molt and was not observed in this study. The most distinguishing feature of the cirri was the state of the setae, the finger-like projections used to capture food during cirral pumping. As shown in Fig. 2a-b, pre-molt samples (stage D) were readily identified by the formation of new setae within the existing ones and invaginations into the main tissue of the cirri. Post-molt samples were noted by the absence of these features and the setal matrix in an expanded state (Fig. 2c-d). Using these identifiable features, the molt stage of each barnacle sample was categorized as being either pre- or post-molt.

### RNA-seq transcriptome analysis

To understand barnacle cement protein gene expression levels in pre- and post-molt growth stages, RNA-seq technology was used to compare transcriptomic profiles of pre- and post-molt barnacles, specifically examining the sub-mantle tissue containing cement gland tissue. We first provide a general summary of these results. Complementary DNA (cDNA) libraries were constructed from three independent samples of sub-mantle tissue collected in pre-molt or post-molt stages. Two additional cDNA libraries were made from two pooled main body samples as pilot experiments for a total of eight separate samples.

Paired-end sequencing technology (Illumina) was performed on each of the eight cDNA libraries. A total of 149,850,897 trimmed high-quality reads giving approximately 13.4 Gbp total sequences were generated with an average trimmed read length of 90 bp. *De novo* assembly of the *A. amphitrite* transcriptome was performed from combined reads from all eight samples using the Trinity program [18, 19]. The summary of transcriptome sequencing and assembly is shown in Table 1. A total of 114.23 Mbp transcriptome sequences were assembled into 163,929 overlapping sequence data reads (contigs) with an average length of 697 bp. A summary of the contig length distribution is presented in Additional file 1. The transcriptome was then annotated with the program Trinotate [18]. From these data, 160,266 transcripts were predicted. Only 37,378 transcripts (23.3 %) have annotated functions that were identified using the SwissProt protein database and 6975 are assigned with gene ontology (GO) terms in 51 functional groups. Abundance profiles of functional groups (Additional file 2) are very similar to those previously reported in *A. amphitrite* and *T. j. formosana* [15, 16].

**Table 1:** Summary of *A. amphitrite* transcriptome

Total reads	149,850,897
Average length of each read (bp)	90
Total assembled sequence size (bp)	114,232,646
GC content (%)	53.24
Contig number	163,929
Contig mean length (bp)	697
Contig N50 (bp)	1202
Predicted transcripts	160,266
Transcripts annotated by SwissPro	37,378
Transcripts with GO term	7045

### Cement protein gene expression: pre-molt versus post-molt

There is strong evidence that cement proteins are required for permanent adhesion of barnacles to their substrate and are secreted by cement glands located in the sub-mantle tissue [2–5, 7]. A number of studies have identified five genes encoding cement proteins (termed cp19, cp20-1, cp20-2, cp52 and cp100 based on their molecular weight) from several barnacles including *M. rosa*, *A. amphitrite* and *T. j. formosana* and these studies also demonstrated most were abundantly expressed in the main body of the barnacle [15, 16]. In this study, all of these cement protein transcripts were identified within the sub-mantle tissue (Table 2). Additionally, a novel paralogue of cp100k, named Aacp114k (*A. amphitrite* cement protein, M.W. 114kD), was also identified. This protein shares 58 % homology with the amino acid sequence of the previously identified cp100k in *A. amphitrite* (accession number: AGS19349). See Additional file 3 for sequence comparison and phylogeny analysis compared to other 100 k homologues in *A. amphitrite*, *M. rosa* and *T. j. formosana*. To confirm the accuracy of transcriptome assembly of the *Aacp114k* gene, the full length cDNA (3 Kb) of this gene was amplified from the sub-mantle RNA with RT-PCR, cloned into the pET22b vector and sequenced. All but six nucleotides from the cloned fragment were identical to the assembled sequence (data not shown), indicating that Aacp114k gene is unique and was indeed present and expressed in the sub-mantle of *A. amphitrite*. Sequence discrepancy between the Illumina sequencing and Sanger sequencing indicate heterogeneity within the samples collected for this study.

**Table 2:** Comparison of cement proteins in *A. amphitrite*

Protein ID	Length (aa)	pI	MW (kD)	GenBank Accession Number
Aacp114k	1000	10.02	114.2	KP863707 (this study)
Aacp100k	1156	10.19	129.5	AGS19349
Aacp52k	629	10.47	73	KP863709 (this study)
Aacp20k-1	113	5.26	12.9	AFX74689
Aacp20k-2	136	8.91	16	AFX74690
Aacp19k	629	9.67	20.2	KP863708 (this study)

**Fig. 3:**
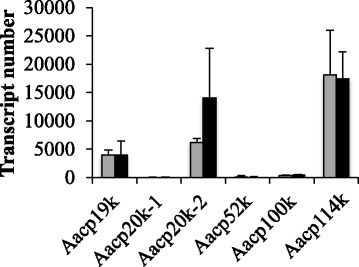
Pre- and post-molt comparison of genes encoding cement proteins. Transcript numbers of cement protein genes in the pre- and post-molt conditions from RNA-seq analysis. Gray and black bars represent pre- and post-molt gene expression, respectively. Values are expressed as mean ± SD from three experimental samples in each condition

The transcriptomic data show that cement protein gene expression levels in the sub-mantle tissue did not have significant differences between pre- and post-molt stages, despite significant differences in the expression levels relative to one another (Fig. 3). These data suggest that expression of genes encoding cement proteins identified in the sub-mantle were independent of molting. Statistical analysis of a perceived difference in the pre- and post-molt transcript number of *Aacp20k-2* showed the difference between these two populations is not significant. In terms of the general expression level of cement protein genes, *Aacp19k*, *Aacp20k-2* and *Aacp114k* were expressed significantly higher than the other three cement protein genes based on transcript counts. It is noteworthy that the *Aacp20k-1* and *Aacp20-2* genes showed opposite expression patterns: *Aacp20k-1* exhibited negligible expression, whereas *Aacp20k-2* was highly expressed in the sub-mantle tissue. Unlike the report by Lin et al. that the *cp52k* gene was abundant in the basis of *T. j. formosana* [16], the expression level of *Aacp52k* in the sub-mantle tissue of *A. amphitrite* was relatively low (Fig. 3).

### Cement protein gene expression: main body versus sub-mantle tissue

**Fig. 4:**
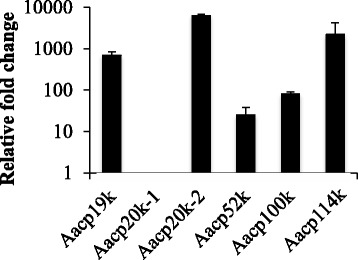
Cement protein gene expression comparison in main body and sub-mantle tissue. Relative fold change (log scale) in cement protein gene expression levels in sub-mantle tissue versus barnacle body. Data are based on qRT-PCR analysis and expressed as mean ± SD from two replicates

Next, the expression level of cement protein genes was compared on the basis of tissue, examining main body versus the sub-mantle tissue of adult *A. amphitrite* samples. mRNA was isolated from the main body and the transcript levels of the cement protein genes measured with qRT-PCR using the cytochrome b gene (*cyb*) as an internal control. In general, the gene expression levels of cement protein genes in the main body were very low as detected by RT-PCR. This accentuates Fig. 4, which is a semi-log plot of the relative fold change in the gene expression level of cement proteins in the sub-mantle tissue compared to the main body. Of the cement protein genes examined, all except *Aacp20k-1* showed varying degrees of increased expression in sub-mantle tissue compared to the main body. The gene for Aacp20-1 was negligibly expressed in sub-mantle tissue and not expressed in the main body. Mass spectrometry was performed on sub-mantle tissue samples processed at the same time as the mRNA collection with the intent to separate the proteinaceous content and target identification of cement proteins. No cement proteins were identified using this technique, though an overwhelming amount of housekeeping and scaffold proteins identified could have masked their presence.

### Secretion analysis

In addition to examining cement protein gene expression levels, material secreted from barnacles at the substrate interface was analyzed using mass spectrometry. There is not a standard method to collect barnacle secretions, so different approaches were employed to identify the presence of cement proteins and attempt to correlate their presence in the sub-mantle tissue and the barnacle/substrate interface. See Methods for detailed explanation of the coverslip, medallion, mortar, and bead collection techniques.

In all secretion samples, the only cement proteins detected with MS analysis were the higher molecular weight Aacp100k and the newly identified Aacp114k (Table 3). Aacp114k was clearly present in the inverted coverslip, medallion, and microsphere samples. Two instances of Aacp114k were identified in the mortar samples. Interestingly, the presence of Aacp100k was confirmed in the medallion and microsphere samples, but not in either the coverslip or the mortar samples.

**Table 3:** Number of unique peptides identified for cp100k

Protein	Coverslip	Medallion	Mortar	Bead
Aacp114k	12^a^	10	2	15
Aacp100k	0	6	0	5

^a^Peptide and protein identifications were accepted if they could be established at > 70 % and 90 % probability, respectively. Protein identifications also had to contain at least 2 identified peptides (see Methods)

### Extended analysis of differential gene expression: Pre- and post-molt

**Fig. 5:**
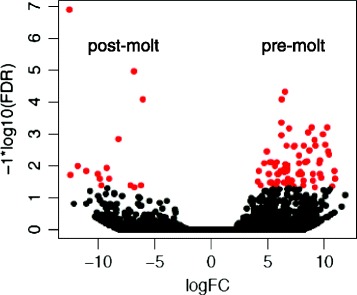
Volcano plot of differential gene expression profile in pre- (right) and post-molt (left) conditions. Data points in red correspond to a false discovery rate (FDR) < 0.1

One objective of this study was to examine the gene expression levels of cement proteins in the sub-mantle tissue and identify cement proteins from barnacle secretions. However, the transcriptome data collected from the pre- and post-molt sub-mantle tissue samples allowed for a more general examination of differential gene expression. The edgeR analysis package [20] revealed that of all predicted transcripts discovered from the transcriptome, 135 were differentially expressed between pre- and post-molt stages (false discovery rate (FDR) < 0.1). A volcano plot representing highly expressed transcripts in the pre- and post-molt samples is shown in Fig. 5. Among these, 111 were highly expressed in pre-molt samples and only 24 transcripts in post-molt samples. These results indicate that barnacle molting is regulated by a limited number of genes with significantly more involved in preparing for and initiating molting.

To confirm the RNA-seq result, 11 transcripts were randomly selected from the differentially expressed transcripts and subjected to real time RT-PCR analysis. (See Additional file 4 for primer sequences and known gene functions). The expression changes between pre- and post-molt were indeed correlated to the RNA-seq data. Annotated transcripts that were differentially expressed in pre- and post-molt samples are listed in Table 4. Several of the highly expressed transcripts in the pre-molt samples were found to encode cuticular proteins, metalloproteases, C-type lectins, and antioxidant enzymes (highlighted in gray in Table 4). Four of the transcripts encoding cuticle proteins and up-regulated in pre-molt samples were found to contain the Rebers-Riddiford (RR) consensus motif (Cx_8_Gx_6_YxAxExGYx_7_Px_2_P) [21], either in its complete form or in the partial form missing the two C-terminal proline residues (Additional file 5).

**Table 4:**
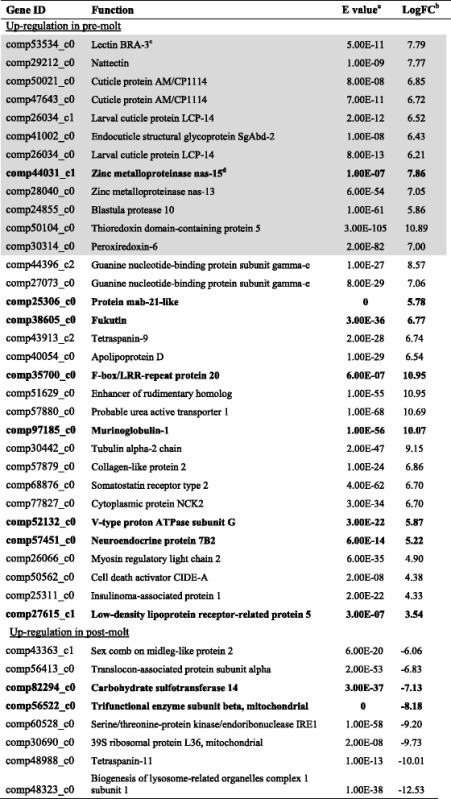
Differentially expressed transcripts in the pre- and post-molting conditions annotated from SwissPro database

^a^ *E* value represents the transcript has the best hit in Swiss-Pro database; ^b^transcript fold change (FC) in log10 between pre-molt and post-molt samples; ^c^items shaded in gray potentially linked to a pre-molt condition and most are discussed in further detail in main text; ^d^genes in bold indicate that their encoded proteins were identified in secretion analysis using mass spectrometry

Of the 135 differentially expressed transcripts in the pre- and post-molt samples (Additional file 6), proteins encoded by 27 of them (20 %) were identified in the secretion samples studied by proteomics, as highlighted in Table 4. Additional file 7 provides detailed information about protein identifications from these samples. Interestingly, one of the encoding genes highly expressed in pre-molt samples, comp97185_c0 (Additional file 6) shares 44 % sequence homology to the settlement inducing protein complex (SIPC) of both *A. amphitrite* [22] and *M. rosa* [23]. Peptides of this protein were recovered and identified in all four barnacle secretion collections.

## Discussion

In this study, we compare gene expression levels of pre- and post-molt barnacles while also examining the main body versus the sub-mantle tissue. The first comparison was enabled by tracking the shed exuvia of several barnacles and collecting sub-mantle tissue at time intervals preceding or following molt. The molt stage was confirmed by examining cirri anatomy via light microscopy and correlated with established stages of barnacle molting [17]. The two comparisons presented in this study have resulted in a thorough analysis of principal genes differentially expressed in pre- and post-molt stages and also a more complete picture of the abundance and presence of putative barnacle cement proteins involved in adhesion to the underlying surface of an adult barnacle.

The assembled transcriptome size (~114 Mbp) in this study is more than four times greater than the number previously reported in *A. amphitrite* by Chen et al. [15]. In RNA-Seq analysis of differential gene expression in different conditions, it is very common that *de novo* assembling of eukaryotic transcriptome often reports fragmented transcripts of one gene or repeated contigs that only differ by a single-nucleotide polymorphism (SNP) or a base insertion or deletion (indel). Such expanded transcriptome size is usually evidence of isoforms of the same gene and artifacts such as sequencing errors, repeats, and variations in coverage and genetics within diploid individuals and pooled samples [24]. In our study, RNA of the pre- and post-molt samples was from six individuals and RNA of the whole bodies was collected from two pooled samples, each containing ten individual barnacles. The transcriptome generated from those eight samples resulted in many paths in the graph construction during the assembly process, effectively fragmenting the assembly which ultimately produced 163,929 assembled contigs. The main contribution to the larger transcriptome size might be heterogeneities within input samples, which was indicated by following evidence: (1) when sequence reads from the pre- and post-molt conditions were assembled separately, the contig numbers were greatly reduced to 73,973 and 70,840, respectively, and the total transcriptome sizes were reduced to approximately 42 Mbp (see Additional file 8). (2) Further clustering these individual assemblies using a homology approach (CD-HIT, 95 % homology) resulted in an overall reduced assembly of approximately 54.8 Mbp in 95,873 contig clusters (35 % reduction from combined). Clustering the individual assemblies results in ca. 60 k contig clusters in each condition, which might be closer to the expected gene number of the barnacle. The fact that the contig sequences from the pre- and post-molting samples do not cluster to a greater degree at 95 % suggested that variation (splicing, SNP, sequencing error) played a greater role in the transcriptome assembly. (3) Finding a six nucleotide difference between the assembled and the cloned *Aacp114* gene sequences (data not shown) also indicated the heterogeneity phenomenon present within individuals. Further inspecting the reads mapping to the *Aacp114k* gene, we observed many differences between the consensus sequences not shared across individual samples (data not shown).

The Illumina sequencing approach employed in the current study produced sequence coverage at ~114x, which is an order of magnitude higher than 8.6x coverage using the 454 method reported by Chen et al. [15]. Higher depth of sequencing would reveal more real alternative splicing transcripts and splicing variants, which contribute to greater numbers of assembled sequences and contigs. On the other hand, higher coverage may also amplify issues with genomic contamination, partially spliced products, and other artifacts of the method. In this study, we were only interested in genes changing within the conditions queried, negating many of these issues in the final differential analysis. As such, reducing the putative transcript/gene (cluster) tally was not necessary. Here, only about 23 % of the ~160,000 predicted transcripts had annotated functions based on available database sequences, meaning the vast majority are unidentified. This trend was consistent even among the differentially expressed proteins in the pre- and post-molt tissue samples, indicating the need for a much larger effort of creating a fully annotated barnacle genome.

The main purpose of targeting the sub-mantle tissue for temporal comparative analysis is that, anatomically, this region is where cement-related tissue of adult barnacles resides. While it was straightforward to separate main body from sub-mantle tissue with little cross-contamination, collection of only cement gland tissue within the sub-mantle cavity proved more challenging. Ovarian tissue, identified by its distinct color and texture, was often intimately intertwined with the surrounding tissue, which included the cement glands [25, 26]. During sample collection, care was taken to remove the vast majority of ovarian tissue, though its complete removal was beyond the scope of this investigation given the limits of dissection. Therefore, potential contamination of a minimal amount of ovarian tissue in the sub-mantle samples is a possibility. Despite this, the aim was to collect as much of the non-ovarian sub-mantle tissue as possible in order to have an optimal sampling of material thought to contain cement precursor materials. Judging by the gene expression data comparison between sub-mantle tissues and the main body, the sub-mantle tissues clearly demonstrates elevated expression of cement proteins and resulted in the identification of a new cement-related protein.

In regard to temporal expression of cement protein genes, there have been previous claims of differential cement protein gene expression as a function of barnacle molting [9, 13], however, these are unsubstantiated and cite unpublished work. In the current study, several barnacle samples with regular molt cycles were chosen in either a pre- or post-molt state accompanied by collection of cirri for molt cycle confirmation based on established criteria [17]. For the detected cement protein genes (Fig. 3), there was no significant difference in the pre- and post-molt gene expression levels despite that fact that several other genes were found to be differentially expressed (Table 4). It may be possible to increase the resolution of molt-dependent gene expression using qRT-PCR according to eight (sub-)stages described by Davis et al. [17]. However, differential gene expression was intentionally limited to pre-molt and post-molt due to the considerable number of other parameters that, collectively, are difficult to control and monitor. These include barnacle size, age, and season, time variability within each molt stage and transcript heterogeneity. Despite these variables, two phenotypes were observed in the present study and were consistent among themselves.

The presence of cement proteins Aacp20-1 and −2 has been reported previously and were shown to localize and express in different types of secretory cells in the cyprid cement gland [27]. In adult barnacles, He et al. reported that protein Aacp20k-1 was present in soft tissue, whereas protein Aacp20k-2 was dominant in the basal shell of adult barnacles, which agrees with our gene expression results (Fig. 4). Interestingly, Aacp20k-1 is the only cement protein with a low *pI* of ~5 (whereas the *pI* values of all other cement proteins are ~9 or greater, see Table 2) and its gene expression was detected at very low levels in the sub-mantle tissue and not at all in the body. It is possible that transcripts of Aacp20k-1 are not stable and were degraded, making it difficult to assess if these results are coincident. Nevertheless, the questions of how and why this transcript is regulated are of interest.

We report high levels of gene expression of a novel paralogue cp100k protein termed Aacp114k. Irrespective of the pre- or post-molt stage, this newly identified protein had the highest gene expression compared to all other putative cement proteins located in the sub-mantle tissue based on transcript numbers (Fig. 3). These results and the relative change in expression level in the sub-mantle tissue versus body (Fig. 4) suggest that this protein is localized in sub-mantle tissue. Proteomic analysis confirms this protein is found in material secreted by the barnacle to a greater extent than the Aacp100k paralogue, as observed in coverslip, medallion, microsphere and mortar samples (Table 3). Based upon our gene expression and mass spectrometry results, we suggest the Aacp114k paralogue identified in the current study is a prominent protein component of barnacle cement in *A. amphitrite*. Interestingly, phylogenetic analysis of the various cp100 homologues show a closer relation between the cp100 homologue in *A. amphitrite* first identified by Chen et al. [15] and in *T. j. formosana* [16] than between the two paralogues found in *A. amphitrite* (Additional file 3).

A previous report on *M. rosa* claimed cp52k, cp68k, and cp100k as being the major protein components of barnacle cement [7]. However, we were not able to identify a cp68k-related gene from the transcriptome in *A. amphitrite* mainly because the cp68k sequence is unavailable in public domains (such as Genbank, SwissPro) despite references to it having been identified and its sequence [9, 13]. Using N-terminal sequences of eight cyanogen bromide (CB) peptides determined from the insolubilized cement fraction of *M. rosa* [8] as queries, we found that only five of those peptides were homologous to either Aacp52K or Aacp100K in our transcriptome database. Therefore, more studies are required to verify whether cp68k exists as single protein in *A. amphitrite* and, if so, whether the expression level is differentiated throughout molting.

In comparing the main body to the sub-mantle tissue using RT-PCR, cement protein genes were predominantly expressed in sub-mantle tissue, which is the expected location of barnacle cement precursor materials. Proteomic analysis of barnacle secretions collected using different techniques indicate that Aacp114k and Aacp100k are secreted by the animal at the barnacle/substrate interface. Of course, the inability to detect other putative cement proteins from the secreted materials does not necessarily mean they are not present. Two factors could contribute to this finding. First, the amount of secreted material is expected to be relatively low since the collection methods used in this study are targeted at newly secreted material over a limited time period with a maximum of 2 weeks and minimum of 1 day. (This is in comparison to collection of an entire, fully-formed cement plaque). In addition, MS analysis may lie outside the limits of detection or may indicate the proteins have been cross-linked prior to enzymatic digestion, escaping detection. Combined, these factors are likely to contribute to the limited detection of cement proteins in the various barnacle secretion collections. Nevertheless, the newly described variations to the collection methods of cement material over relatively short time frames using four different approaches (the coverslip, mortar, medallion, and bead techniques) allowed for proteomic comparison with sub-mantle tissue and offer a fresh means of analyzing recently secreted material and newly formed barnacle cement.

We expand upon the insights gained from cement protein (gene) detection and analysis to the more general findings of differential gene expression in the pre- and post-molt samples of the sub-mantle tissue. Most highly expressed genes were found in pre-molt samples and, of those identified, generally encoded proteins in four categories: cuticular proteins, metalloproteases, C-type lectins, and antioxidants. These categories can be associated with molting, biomineralization, morphogenesis, and cell adhesion. Here, we provide a more in-depth discussion of some of the highly expressed protein genes identified in the sub-mantle tissue of pre-molt barnacle samples.

The increased expression of cuticular protein genes was not unexpected since molting requires significant production of cuticular material and tissue scaffolding to replace the soon-to-be-shed exuvia. Several cuticle-related protein genes in the pre-molt samples were identified and four were found to have the chitin-binding Rebers-Riddiford consensus motif [21]. Multiple transcripts of RR-containing cuticle proteins were reported to be temporally and spatially expressed in the decapod crustacean *Callinectes sapidus* and involved in biomineralization [28]. More than 60 homologs of this type of cuticle protein gene were identified from the transcriptome data in this study, indicating a variety of functions at different growth stages or conditions. RR-containing cuticle protein genes highly expressed in the pre-molt sub-mantle tissue of barnacles strongly suggest an involvement with chitin and possibly a role in biominerialization, both of which have a role in barnacle growth [4].

Two genes encoding C-type lectins, BRA-3 and nattectin, were also found to be highly expressed in the pre-molt condition. C-type lectins usually bind carbohydrate structures in a Ca^2+^-dependent manner and are well-recognized immune response initiator proteins [29]. They have been found to participate in mineralization [30] and inhibit the growth of calcium carbonate crystals [31]. High expression of these two genes in the pre-molt condition suggests that they have significant roles in biomineralization during growth/molting and coordinating post-molt responses.

Coincident differential expression of genes encoding peroxiredoxin-6 and thioredoxin domain-containing protein 5 in pre-molt samples suggests that the barnacle experiences oxidative stress during or following molting, since peroxiredoxin uses thioredoxin to recharge after reducing hydrogen peroxide (H_2_O_2_) so as to restore its catalytic activity [32]. Antioxidant proteins like peroxiredoxin are often coupled with others (like thioredoxin) to limit damage of oxidative stress to host tissue [33].

Increased expression of genes encoding proteases (including zinc metalloproteinases) in the pre-molt stage points toward proteolytic activation of proteins, including contributors to barnacle cement, having an essential role for effective cement cross-linking and co-adhesion within the interface. Zinc metalloproteinases have a significant role in inflammation as enzymes that rework extracellular matrices and remodeling of structural material [34]. In addition, it has previously been suggested that barnacle cement cross-linking involves proteolytic activation of structural proteins in order to maximize the potential for bonding interactions with other proteins and with the surface [35].

A glycoprotein, settlement inducing protein complex (SIPC) has been reported as the species-specific cue to settlement of barnacles [22, 23] and expressed in the cuticle of adults [36]. It has been reported as a complex of three proteins (98, 88 and 76 kDa) but retains some biological activity as separate subunits [37, 38]. In the transcriptomic data, we found SIPC that is 100 % identical to the previously reported one [22] and its gene expressed at similar levels in both pre- and post-molt conditions. Interestingly, we also found a homologous SIPC gene, comp97185_c0 (Additional file 6), highly expressed in the pre-molt condition and its encoded peptides were identified in all secretion samples analyzed with MS. This finding suggests that the barnacle may secrete a new and different type or combination of SIPC molecule with alternative (non-SIPC) ligands as a molting signal, and its function is worth further investigation.

## Conclusion

This work advances the understanding of the complex process of barnacle molting/growth by defining the temporal and tissue distribution of various proteins, including those reported to be major components of barnacle cement. Our analysis suggests there is not differential expression of cement protein genes in pre- and post-molt barnacles and most appear to be highly expressed in the sub-mantle tissue compared to the main body. In addition, we identify a novel cement protein homologue, Aacp114k, with high gene expression levels in the sub-mantle tissue and confirmed its presence in secretions at the barnacle/substrate interface. In the absence of a fully annotated genome, our transcriptomic analysis reveals highly expressed genes identified in pre-molt barnacle samples and paints a picture of extensive cuticular development, biominerialization, and proteolytic activity that may be associated with the precursor elements of barnacle cement. Our results also highlight the fact that the majority of differentially expressed genes in the pre- and post-molt stage are unidentified and potentially unique to barnacles.

The description of several new cement collection techniques opens opportunity to build a profile of the macromolecules present and active at the barnacle/substrate interface with the goal of elucidating the mechanism of the cementing process. Future directions include developing approaches to collect and profile pre-secreted material as well as establishing protocols to identify the location of various cement components prior to secretion. We note that *in situ* hybridization studies have been carried out in *A. amphitrite* cyprids [17] and offer great potential for identifying the tissue (or cell) specific expression of particular genes relevant to the current study. Given the increased complexity of the adult barnacle anatomy and the fact that all tissue is surrounded by an opaque calcified shell, there are challenges with *in situ* hybridization that are beyond the scope of the current work yet are a promising future direction.

Ultimately, the research goals are to identify the various precursor components of barnacle cement, understand the mechanism of delivery, and how the components interact at the substrate interface. A complete understanding of this process can lead to targeted strategies to mitigate barnacle adhesion as well as mimic its functionality in underwater environments.

## Methods

### Barnacle husbandry

*A. amphitrite* cyprids were settled and raised on silicone coated panels as previously described [39] until they were mature enough to be fed *Artemia* spp. nauplii. Samples were then shipped to the Naval Research Laboratory where they were maintained in an incubator at 23 °C with 12 h daylight cycles in 32 ppt artificial seawater and fed *Artemia* spp. nauplii every other day. In addition, the water for barnacles was changed weekly during which the algal growth was removed. Barnacles were reattached from the silicone panels to alternative substrates for the experiments [40].

### Molt cycle analysis and cirri imaging

A subset of over ten barnacles were reattached to glass slides, kept individually in separate containers to track the molt cycles over the course of several weeks (minimum of 2 weeks). Molting was noted by the presence of the exuvia in an individual barnacle container and, depending on the age or size of the barnacles, occurred anywhere from every 3 to 7 days. Barnacles molting in a regular pattern were selected for this study. For pre- and post-molt comparison, three barnacles were sacrificed the day prior and three the day after a molt, for a total of six individual samples. The cirri were removed for light microscopy observation (see below). In other barnacles undergoing regular molt cycles, only the main body was collected for subsequent protein and mRNA analysis and the cirri were removed for light microscopy analysis.

Cirri were examined using differential interference contrast (DIC) light microscopy on a Nikon TE2000 inverted microscope to confirm the molting stage of each sample. Following the procedure of Davis et al. [17], setae from the upper portion of cirri (rami) were imaged under 20× and 40× magnification. Several segments were imaged for consistency and structural features identifying the molt stage were noted. Davis et al. categorize four main stages, A through D, of barnacle molting proceeding through a short time period following molting (stage A) to the period prior to molting (stage D). These main stages are discriminated further into eight sub-stages. For the purposes of this study we have generalized these stages to two main categories: i) pre-molt (proecdysis, stage D) and ii) post-molt (post- or interecdysis, stages B or C).

### Total RNA and protein collection

Isolation of total RNA from whole body tissue without the sub-mantle tissue was performed in the following manner. First, the main body was removed from a barnacle shell that had been cracked open with sharp tweezers followed by the addition of TRIzol Reagent (Life Technologies Inc., Grand Island, NY) according to the manufacturer’s protocol. The soft body tissues in TRIzol were vigorously vortexed and resuspended several times to break them apart. Chloroform was then added and mixed evenly by vortexing. The mixture was allowed to sit at room temperature for 2–3 min and centrifuged. Subsequently, the liquid phase was removed without disturbing the interphase precipitate and mixed with isopropanol to precipitate total RNA, followed by centrifugation and washing the pellet with 75 % ethanol. The rest of liquid phase including the interphase precipitate was then subjected to DNA and protein isolation according to the manufacturer’s protocol. The total RNA was further treated with DNAse I to get rid of DNA contaminants using on-column digestion (Qiagen Inc., Valencia, CA).

A similar procedure was used for isolating total RNA from sub-mantle tissue. The main body of the animal was removed as well as the underlying inner mantle and the sub-mantle tissue carefully collected with several gentle pipette suctions for processing. A schematic of the barnacle anatomy highlighting the main body and sub-mantle tissue is shown in Fig. 1. Ovarian tissue, which was clearly evident based on its color and texture with respect to surrounding tissue, was carefully removed with tweezers to minimize/eliminate its inclusion for subsequent processing/analysis.

For all samples, isolated total RNA were examined by agarose gel electrophoresis. A quality check was performed using a Bioanalyzer (Agilent Technologies) and RNA samples were found to be of high quality with no DNA contamination.

### mRNA sequencing and data analysis

Three separate samples of the sub-mantle tissue from the pre- and post-molting conditions and two pooled samples from the whole body (10 samples in each; all without the sub-mantle tissue) were collected for RNA sequencing (RNA-seq) experiments. Enrichment of poly(A) RNA, synthesis of cDNA and construction of cDNA libraries were processed using protocols provided by Illumina, Inc. (San Diego, CA). Libraries were sequenced on an Illumina HisSeq2000 Sequencer in 100 paired-end configuration. Following sequencing, data was trimmed for both adaptor and quality using a combination of EA-utils and Btrim [41, 42]. Trimmed-paired reads were then *de novo* assembled using Trinity software using the default parameters found in the online protocol [18]. Assembled transcripts were also further clustered into putative gene clusters using CD-HIT-EST [43]. Annotation of transcripts was performed using the Transdecoder/Trinotate package [http://www.vcru.wisc.edu/simonlab/bioinformatics/programs/trinity/docs/annotation/Trinotate.html] (blast based annotation using Swissprot) with the default E-value as 1e-6. Read alignment and estimation of transcript abundance was performed using Bowtie2 [44] and RSEM [45] as described in the Trinity abundance estimation protocol. Transcripts differentially expressed between two conditions were identified by comparing absolute number of transcripts across biological replicates with FDR < 0.1 as the cutoff value using the EdgeR software package [20], again as described in the Trinity protocol. Raw sequence reads were deposited in Genbank as BioProject PRJNA271096.

### qRT-PCR

The primers used for the quantitative real-time reverse transcription polymerase chain reaction (qRT-PCR) assays are listed in Additional file 4. The cytochrome b gene (*cyb*) of *A. amphitrite* was chosen as an internal control. The qRT-PCR was conducted on an iCycler (BioRad Laboratories) using the iScript OneStep RT-PCR Kit with SYBR Green (BioRad Laboratories). qRT-PCR reaction mixtures consisted of 10 ng of total RNA from each extracted sample, 200 nM of primers and were subjected to the following cycling conditions: one cycle at 50 °C for 30 min and 95 °C for 15 min, followed by 40 cycles of 94 °C for 15 s, 52 °C for 30 s and 72 °C for 30 s. Triplicates for each RNA sample were collected for statistical analysis. The average cycle threshold (Ct) value for each tested RNA sample was obtained and the gene expression level was then used for comparison among tissue samples. Relative quantities of transcript were determined using the 2^-ΔΔCt^ formula. ΔCt is the difference in Ct of the gene of interest and Ct of the normalizer gene, and ΔΔCt is the difference in ΔCt from the sub-mantle sample and ΔCt from the body sample. Cytochrome b gene (*cyb)* was used to normalize expression levels of genes of interest because its transcript was constant in all conditions in this study.

### Cloning and sequencing of Aacp114k encoding gene

Total RNA isolated from sub-mantle tissues were reverse transcribed to cDNA according to the manufacturer’s protocol (Life Technologies). The first strand cDNA was then used as a template along with the primers, 114KNcoIF (5′AAAAACCATGGGCATGCTGCGGCTCTCGCTAGC3′) and 114KXhoIR (5′GGGGGCTCGAGGCACTTGAAGTAGTCGTAC3′), to amplify the Aacp114k transcript by conducting PCR. Resulting 3 kb DNA fragments were digested with *Nco*I and *Xho*I, followed by cloning into a pET22b vector. The cloned plasmids were propagated in *E. coli* Top10 in LB culture supplemented with ampicillin. The insert of isolated plasmid was sequenced using the Sanger sequencing method (Eurofins Genomics).

### Barnacle secretion collection

There is not a standard procedure to collect barnacle cement and secretions, so to maximize the opportunity to identify potential cement proteins collection of barnacle secretions was carried out in three different ways to produce four different samples for subsequent analysis. From each collection method, the majority of protein was solubilized. Any insoluble fraction was minimal and remained on the respective surface. A description of each technique is provided below.

### Coverslip

In the first, adult barnacles were gently removed from silicone substrates and all shell plates, including the base plate, were rinsed and cleaned with a cotton swab in deionized water. The barnacles were then inverted and settled onto a Plexiglas® platform engineered with circular openings, which allowed the apical portion of the organism to be partially submerged in sea water to allow for continuous feeding and growth during the cement collection process. A No. 2, 12 mm diameter borosilicate cover glass (Fisher Scientific #22-293-232) was placed on top of the exposed base plate as a substrate to collect secreted material from the barnacle (see Additional file 9 for schematic of setup). After 24–48 h at room temperature, the cover glass substratum was gently removed using a dissecting needle. In order to maximize sample collection, the entire cover glass was placed into a 1.65 mL microfuge tube and crushed with a glass rod. Next, 100 μL 1× Laemmli sample buffer was directly added to the sample and heated for three minutes at 95 °C. Finally, 50 μL of the solubilized sample was separated by SDS-PAGE (4-20 % TGX precast gel; BioRad) and used for further mass spectrometry analysis. These samples are referred to as “coverslip” samples.

### Mortar and medallions

In the second, adult barnacles were transferred from silicone panels and settled on sodium aluminoborate (Na_2_O • Al_2_O_3_ • 3B_2_O_3_) glass substrates, which form a hydrated reaction layer (<25 μm thick) in aqueous environments that is resistant to barnacle adhesion [46]. After 2 weeks, barnacle bodies and side plates were carefully removed, leaving the base of the barnacles attached to the substrates which were then cleaned with a cotton swab in deionized water to remove loosely bound organic matter (see Additional file 9). Base plates were then de-minerialized by immersing substrates in 0.1 M ethylenediaminetetraacetic acid (EDTA) at room temperature for 48–72 h. The organic remnants of the base plates were collected and placed in 50 μL of 0.1 % (w/v) sodium dodecyl sulfate (SDS) solution in deionized water for subsequent analysis; these are referred to as “mortar” samples. Likewise, barnacle “medallions,” consisting of the cuticular layer and underlying barnacle secretions, were gently rinsed with deionized water then peeled off the aluminoborate glass substrates and placed in 50 μL of the SDS solution. Next, 50 μL of Laemmli sample buffer containing 300 mM dithiothreitol (DTT) was added to both samples and the resulting suspensions were incubated for 15 min at 95 °C to solubilize proteins in the samples. After centrifugation (9000 rpm for 1 min), supernatants were separated by SDS-PAGE for further analysis. This procedure resulted in the cuticle being pelleted to the bottom of the centrifuge tube.

### Bead

In the third type of preparation, borosilicate glass microspheres (<50 μm in diameter) were placed in the bottom of dishes filled with ASW to form a bed of microspheres 2–3 mm in depth. Adult barnacles (*n* = 5) were then transferred from silicone panels onto the bed of microspheres. After 24 h, barnacles were lifted out, and those with microspheres attached to their underside (three out of the five) were gently scraped to remove the majority of the microspheres without damaging the barnacles (see Additional file 9). The collected microspheres were placed in 50 μL of an SDS solution, vortexed, and centrifuged at 9000 rpm for 1 min. The resulting supernatant was collected, mixed in a 1:1 ratio with Laemmli sample buffer, and then separated by SDS-PAGE for further analysis and referred to as “bead” samples.

### LC-MS/MS

Individual bands from protein extracts of each sample separated on SDS-PAGE gels were excised and digested in gel by trypsin. Peptides were extracted by 2 % formic acid in 50/50 acetonitrile/water, followed by 100 % acetonitrile. Digests were analyzed by liquid chromatography-mass spectrometry/mass spectrometry (LC-MS/MS) using Tempo-MDLC coupled to a QStar Elite mass spectrometer (AB Sciex, Foster City, CA). Tandem mass spectra were extracted by AB Sciex MS data convertor version 2. Charge state deconvolution and deisotoping were not performed. All MS/MS samples were analyzed using Mascot (Matrix Science, London, UK; version 2.4.1) and X! Tandem (The GPM, thegpm.org; version CYCLONE (2010.12.01.1)). Mascot was set up to search the BALAM_adhesproteins_003 database (1492 entries) assuming the digestion enzyme trypsin. X! Tandem was set up to search a subset of the BALAM database also assuming trypsin. Mascot and X! Tandem were searched with a fragment ion mass tolerance of 0.80 Da and a parent ion tolerance of 0.80 Da. Deamidation of asparagine and glutamine, oxidation of methionine, acetyl of the N-terminus and carbamidomethyl of cysteine were specified in Mascot as variable modifications. Glu- > pyro-Glu of the N-terminus, ammonia-loss of the N-terminus, gln- > pyro-Glu of the N-terminus, deamidated of asparagine and glutamine, oxidation of methionine, acetyl of the N-terminus and carbamidomethyl of cysteine were specified in X! Tandem as variable modifications. Scaffold (version Scaffold_4.2.1, Proteome Software Inc., Portland, OR) was used to validate MS/MS based peptide and protein identifications. Peptide identifications were accepted if they could be established at greater than 70 % probability by the Peptide Prophet algorithm [47] with Scaffold delta-mass correction. Protein identifications were accepted if they could be established at greater than 90 % probability and contained at least 2 identified peptides. Protein probabilities were assigned by the Protein Prophet algorithm [48]. Proteins that contained similar peptides and could not be differentiated based on MS/MS analysis alone were grouped to satisfy the principles of parsimony. Proteins sharing significant peptide evidence were grouped into clusters.

### Availability of supporting data

The data sets supporting the results of this article are available in the Proteomics IDEntifications (PRIDE) repository, http://www.ebi.ac.uk/pride/archive/projects/PXD001982. Other supporting data are presented in Additional files 1, 2, 3, 4, 5, 6, 7, 8 and 9 and also found in the same repository.

## Additional files

Additional file 1:
**Plot summarizing the contig length distribution.** (PDF 92 kb) Additional file 2:
**Graph with the abundance profiles of functional groups from the combined pre- and post-molt data sets.** (PDF 73 kb) Additional file 3:
**Sequence comparison and phylogeny analysis of Aacp114k compared to other 100 k homologues in** ***A. amphitrite***, ***M. rosa*** **and** ***T. j. formosana***. (PDF 213 kb) Additional file 4:
**RT-PCR primer sequences of 11 transcripts randomly selected from the differentially expressed transcripts and their gene functions.** Primer sequences of the cement proteins and cytochrome b are also included. (PDF 17 kb) Additional file 5:
**ClustalX alignment of deduced cuticle protein sequences which encoding genes were highly expressed in the pre-molt stage.** Rebers–Riddiford (RR) consensus sequence is highlighted. (PDF 183 kb) Additional file 6:
**Table of the 135 differentially expressed transcripts in the pre- and post-molt samples.** (XLSX 31 kb) Additional file 7:
**Detailed information about protein identifications from the various secreted samples.** (XLSX 117 kb) Additional file 8:
**Comparison of sequence assemblies in the pre- and post-molt samples.** (PDF 6 kb) Additional file 9:
**Schematics and experimental conditions of the barnacle secretion collection methods.** (PDF 537 kb)
