# Supplementary material for: Molt-dependent transcriptomic analysis of cement proteins in the barnacle Amphibalanus amphitrite
Source: BMC Genomics. 2015 Oct 24;16:859. doi: 10.1186/s12864-015-2076-1 (PMC4619306; doi:10.1186/s12864-015-2076-1)
Supplement: Additional file 1: — Plot summarizing the contig length distribution. (PDF 92 kb) [file 12864_2015_2076_MOESM1_ESM.pdf]

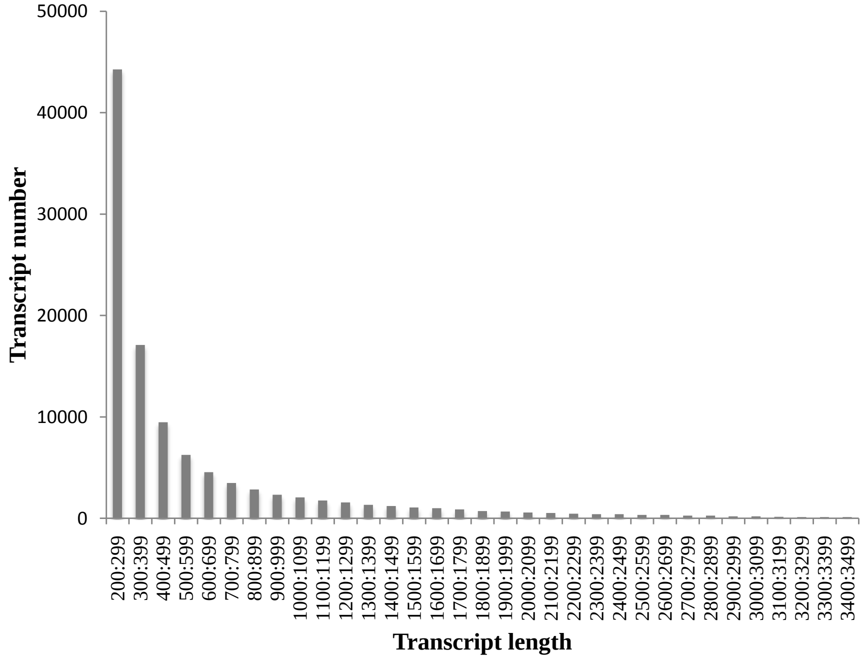

Additional File 1. Contig size distribution of assembled transcriptomes combined from the pre- and post-molting stages.
