# Supplementary material for: Molt-dependent transcriptomic analysis of cement proteins in the barnacle Amphibalanus amphitrite
Source: BMC Genomics. 2015 Oct 24;16:859. doi: 10.1186/s12864-015-2076-1 (PMC4619306; doi:10.1186/s12864-015-2076-1)
Supplement: Additional file 2: — Graph with the abundance profiles of functional groups from the combined pre- and post-molt data sets. (PDF 73 kb) [file 12864_2015_2076_MOESM2_ESM.pdf]

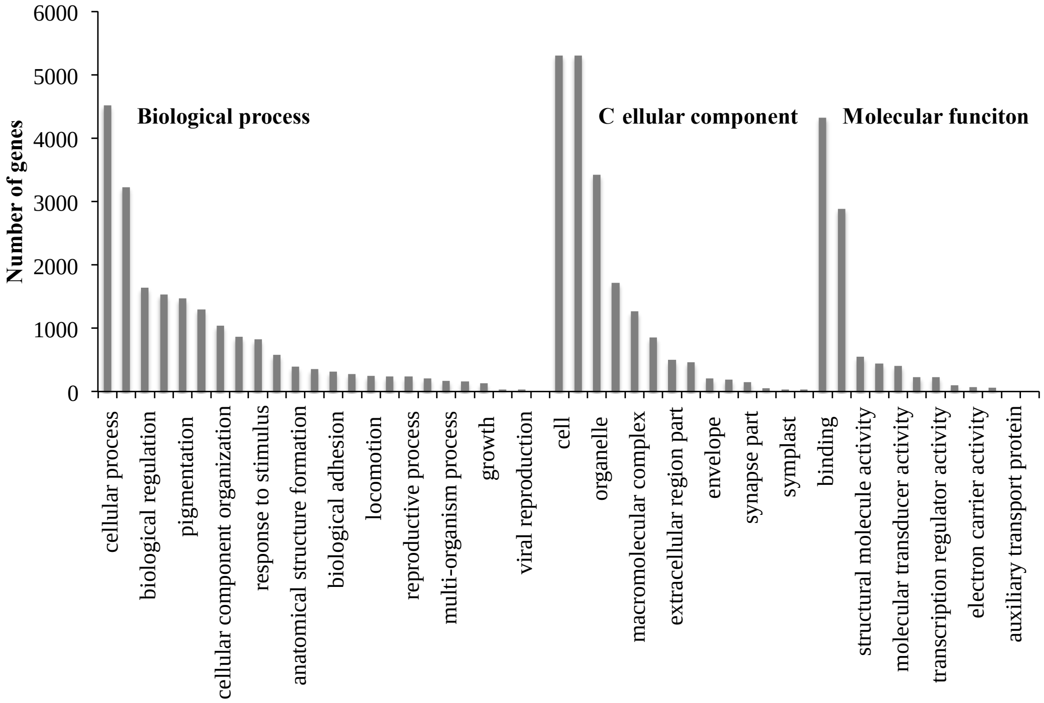

Additional File 2. GO classification of assembled transcriptomes combined from the pre- and post-molting stages. Annotated genes are classified in three main categories: Biological process, Cellular component, and Molecular function.
