# Supplementary material for: Molt-dependent transcriptomic analysis of cement proteins in the barnacle Amphibalanus amphitrite
Source: BMC Genomics. 2015 Oct 24;16:859. doi: 10.1186/s12864-015-2076-1 (PMC4619306; doi:10.1186/s12864-015-2076-1)
Supplement: Additional file 3: — Sequence comparison and phylogeny analysis of Aacp114k compared to other 100 k homologues in A. amphitrite, M. rosa and T. j. formosana. (PDF 213 kb) [file 12864_2015_2076_MOESM3_ESM.pdf]

A)

```
Aacp100k    1 MLRLPLALALLLAASAYG-NVLFARSGCGCLRNPVAAKLTGEEISHLRGYVKERGVKHYD
Tjcp100k    1 MMRVPLALTLLLAASASG-SIMFPRNGCGCLRNPVAAELKTEEITQLRGYIKQRGVTHYE
Aacp114k    1 MLRLSLALAVLLAVSAAGDKYPISRFGCGCNRNIIAADLTVQEISQLRVYIQQLGIKNHP
Mrcp100k    1 MMRLSLVAVLLVTVSVTGHRPSFERRCCGCLRSPVAADLDDDEIGMLREYVKKQGVMHYE

Aacp100k   60 VLSNDALQAIFRFNLINNFPDVVPATRTGVLQIISESLNTLTDAVVPSVPQCGKIAGYLQ
Tjcp100k   60 AFTDYSLQAIYRFNLLNNFPDVVPSSRSGVIQVVSESLKELTDEVVPSAPTCGKID-YLQ
Aacp114k   61 VLTDDVINAIFRFNLNNNYQGQVPSKRSTLLQIFSESFSSLTDAVLPSVSQCGKYGDYLH
Mrcp100k   61 SLSDISLKAIFRNKLLNNFPEEVPATRDGVLQVITESLGSLTDSVVPSVSQCGQIAGYLQ

Aacp100k  120 KSVPGLAAGGVSLDLRSLVASASVILHQRGVTVNLEQLNVLLKSGLAGYLQSTAYQSSYG
Tjcp100k  119 GAVPELAGGGVNIDLKSLVA-ASVILHQRGVAIQLSQLNILLKTGLSGYLZSTAYKTSYS
Aacp114k  121 KATPLVISGDRKFDMCSLVASYAVILHQREVTVNFDQLNVILKLGLQKYLKSTAYQSSYS
Mrcp100k  121 KSVPALAQGGFNVDLKSLVSSASVLLHQRGVTVNTDELNIFLKYGLINYLKSTVYQSSYS

Aacp100k  180 SLIQLISALDHIDHNLPNILDQESLIVVRRALESRFNLDREIFDKRYKLAIKAFEENRRR
Tjcp100k  178 ALSQLIATLDHIDHNLPNILDQDLLIAVRRKLETRFNLDSKIFDQRFQQAIRVFEANRQR
Aacp114k  181 MLTQLLTSLDFIDHDLPTILDYEELIAVRRALLLRYNIKRARFDNRFRLAIEEFKLNRHR
Mrcp100k  181 MLRQLIVTLDYLDHELPVILDYEELIAVRLALKKKFDTSVDIFKNRYQLAIQSYKANRNL

Aacp100k  240 LLASFNTLAYRGPNYETNVQLVIKQMLTIFSGISAKTVRIILNILQLTNSAGGKATPKDL
Tjcp100k  238 ILESFNSLAFRGPDYEVTIQVVIRETIRLFPGISKTTLRSVLNILQLTNTRGGKATPKDL
Aacp114k  241 LLSTFNTIAFRGPYYEIVVQEVIREIIKIFPGLSASSVRIILDVLQLTNAPGGKASPRDL
Mrcp100k  241 LLDSFRTMAYRGPKYEMYLQEAIRETINIFPSISPSTVRIVFNNLQLSNTGSGMVSPLDL

Aacp100k  300 LAMITVPKLDVSIRKITEAAANRVYLKLPEHHQGLTIDDIQEAYSIFIIGLASQGVQPLQ
Tjcp100k  298 LAMITIPHLDKSLRTITDVIANRIFLKLPYHHKGLTRVEVHEAYVLFIIGLASQGVQPVQ
Aacp114k  301 LALITVPRLDAELYVIQQYYIQKYVASLVTYYPSISVEIIKEAYPTFLISLISQGIQPVN
Mrcp100k  301 LAMVTTPVLDDDLKSITKVYAERLYNKMPGCYMG-QEVEIQEMYFLFLVGILSQGIQPLN

Aacp100k  360 LEATYEAFIWHTQRFFLATRIYSVQAYLLYVMRVVVPLIPRGSQSFRLHIFDSSVVIDNI
Tjcp100k  358 LQACHEAFVWHTQRFFLGTRSYTVEAYILYVIRVVVPSIPRGSVGFRLHLFDASIVIDNV
Aacp114k  361 TIVTYKTFYYYLQAYFQSTSSYSVESMTTFFLRTVVTSIPRGSPHFRINIFQSTVVIDNI
Mrcp100k  360 QMAIYELFIYHSTIYFRSSCAYTVDDYFLFISRVVRPNIPLGSKHFKIISFDHSVVIENI

Aacp100k  420 LVPEGLTSIYEEGRQTIIKRIRGLQGSSSDITNRIIGGQGEKGVIGNDLKFQT-IVPADV
Tjcp100k  418 LVPEPLQSIYKEGRQTIIERVRGLQGSSEDITRRLLTGEGEKPFVKNIVDLRPPIKTGPL
Aacp114k  421 LVPQPWTSIYRKGKASIIKRIVGPQGNSRNIIIRLKTGRGEKPVIQNDLKFKN-IVPADV
Mrcp100k  420 LVPEPWRSNYEKSRDTIIKRLVGLQGSSDQITKRLIEGGGEKGYIKNIVNLKPAITPRPQ

Aacp100k  479 PGYDQFEYQNVILSAVQMREIASVLIQRFNQLKQPSLQLPLMRIMIHANVIPNSGAAAAA
Tjcp100k  478 PTYSKFEYEGVILSVAHLQAIAYELQRRFDQLKQPQLQLPLLRVLIRAHVVRGSGDKAAA
Aacp114k  480 PGYDQFEYQNVILSAIQLSQVASALIQRFNLLKQPSLQLSTLRIMIRAGLIKGTGVQAAN
Mrcp100k  480 PTYDAFEYQNVLLSSQHMQRVAFELAKRFEGLKEPSFRLPLLKILVRANLVTDTGDKAAA

Aacp100k  539 AFRRLFRGLPAYSGPTDLSFVLTQLSENRLQLTETQLLAGIQQFYVASRCLGYVIPQQTI
Tjcp100k  538 AFRRLFSGLPRYVAPQDVAGIITQLSERRLQLTRTQVLAGLQQFFVASRCLGHVIPPKSL
Aacp114k  540 AFSTLFQGLPAYSLPTDLTFVFSQLTELNLQLTETQIRGALQQFYVVTRSLGYVIPQETI
Mrcp100k  540 AFLRLFQGLPVFSRPSSLSFIVEQLREYRLQTTKAQIKAALDQFFVATKCLGYVIPQQKI

Aacp100k  599 PSVFLYTVREYLSTLASVPAQPFGDGFLEFLYLRLAGIIRQVTVVDQKVPIDDYVSQKIF
Tjcp100k  598 PGVFVYTITQYIQTLSKIPAQPFDYRFLQYLYQRLASIIQQVVLINRSVPVVGRQVESIF
Aacp114k  600 HSVFVYSVREYLSTLTSIPTQPFGDGFLEFLYLRLEVIIKKVVVIEQQVPIDDYVTQQIL
Mrcp100k  600 PSIFLATVGRYLSTLPTIPKQPFDYNFLEYLRYSLASIIEHLPAVGSQSVIDDYAMYKIF

Aacp100k  659 SVFGSSVRISVEARRTIIRFIHNSELLPKVGQG-VSVVAQYQRLLKSLFKRYPIGTFILS
Tjcp100k  658 SVFG-RVRISLRCQRTIVRFIDNSGLIKRPMKGSSTAVVVYRQLLATMLKRYPVGVFVLS
Aacp114k  660 SVFA-NVRISVEARRTIIRFIHNSKLLPKPQKG-VSVVSQYQALLTSLTKRYPLDIFVLN
Mrcp100k  660 SIFG-HTKLSIYAKRIIIKYINEYKLLPKAAQN-VPLLVQYQQLMESMVSKCSVSSFILS
```

```
Aacp100k  718 TKELVYIRAELK-KAGISVDIKYLRDANVMAYIGLGLLNRLEKSMTVIRVRQIVLSSIRY
Tjcp100k  717 EKELTILRVELSTKYRISITTQYLRDANIMSFVGLGLLGRLKPSLTVVQYRQVLVVSIRS
Aacp114k  718 KNILVQIRSQLI-SAGIQIQLKVLRDINIIAYISLGLMDRLKGQNTVGYVRQIVYSSIRY
Mrcp100k  718 KKQLTTIQSDLYKSRRIRIELSLLVDINYMAYFAVCQSG-----AYTAVMNRYVYQSIIS

Aacp100k  777 FLRINKVSNIPSVEFFRVLLHQYKVPLPQLPLPKRPVIQYPK-YTRAPIYILSGISLPVK
Tjcp100k  777 FLRINKVSNIPTSDYFRVLFRTQHVKTPSLPVPQPPVIQTPKVYVPPPIYILRGLTLTVV
Aacp114k  777 FLRTNKVTSILSLEFVQFLLHKYKVPSPPQ-IWWQPPVEQHKVNIQRDIYILPDIYLPVR
Mrcp100k  773 YTQTVRKPNYYSAEFFRILVESSKG--SKLPVSRPPLVQYRR-TPKRLCYIVPGIVLYRE

Aacp100k  836 QVEQLVVILRTRFVFVSIENVQSILAHTVLLLRASGQQIVQKNCYEVLTRYYRGLPKSIS
Tjcp100k  837 QVREIVAVLRVRFTFVSLDNVQAILAHTVLLLRANGKPVDQKNAYEVLSSYYSSLSKNLA
Aacp114k  836 SVHQLVVILQKRFVFVSIDNVQTIIVHTILILRANGVTITSDNCYDYLYRYYSGLPANIG
Mrcp100k  830 QLRQLVTLIRPRFTFVSMRNIRSIVAHTILILRAR-YSITQNNCYGHLTKYYNGIPIN-A

Aacp100k  896 VGEFDIEDLVKEIDDQLKDATISGTGVQSALVELYLHMYYLKMPLPS-VKVRDGFLSFVI
Tjcp100k  897 ISGVDIDALLKTID-RLVDATISGTGIQSGLVELFLHMSFLKMPIPG-PEVRNEFFSFCI
Aacp114k  896 VESFDISSVLDSIKRYAAQTTVTEVHVQSALVELCIHLYSMELPLPS-VQYRNKFLGYVI
Mrcp100k  888 LGAFDAYNLLQTLRVQPKRAAISSVGIQSAMAELYMHMRHLQMPFPSDNDVRITVLRNCL

Aacp100k  955 GAYGKVQVRRQLPFGKLFYDFLQGFLPKLPGYLKPFPIFAGPQVYKVFHSTLKTPVYPSD
Tjcp100k  955 GAYGQVQVRRQLPFGKSFYEFLSGFLP---------------------------------
Aacp114k  955 DAYGKKYRRHGLPLGARFYKFLKKFLPKAR-HTKPKYISCKYDYFKC-------------
Mrcp100k  948 SAYSSKGMYRNVPFGRRFFAFLNTYLPMRRTAPNKRCMRYKKSFRC--------------

Aacp100k 1015 IPLYFQLFRRVTKGSLTMGSLQSSLSGLSLLPGLTSEELSSIIDLVKGKQLKVSQTEIRR
Tjcp100k      ------------------------------------------------------------
Aacp114k      ------------------------------------------------------------
Mrcp100k      ------------------------------------------------------------

Aacp100k 1075 AFAICRLSLGLSSVKISRSKLISIFQEVVISIVQKYKSLLVVSYVEQILLRIRTYGPKYR
Tjcp100k      ------------------------------------------------------------
Aacp114k      ------------------------------------------------------------
Mrcp100k      ------------------------------------------------------------

Aacp100k 1135 PVQPITPIKPGYPCKNSKYIRC
Tjcp100k      ----------------------
Aacp114k      ----------------------
Mrcp100k      ----------------------
```

B)

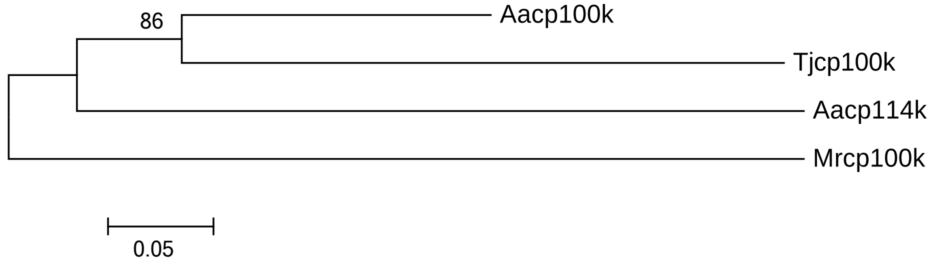

Additional File 3. Comparison of protein sequence of ~100kD cement protein. A) ClustalX alignment of ~100kD cement proteins from three barnacle species; B) Phylogeny analysis of the same cement proteins with Neighbor-joining method using MEGA5 software (Tamura K et al., 2011, Molecular Biology and Evolution 28: 2731-2739). Bootstrap percentage > 50% from 1000 simulations, *i.e.* 85, is shown to the left of branch point. The scale bar represents the amino acid substitutions per site. Aa: *Amphibalanus (=Balanus) amphitrite* (100k-1: AGS19349), Mr: *Megabalanus rosa* (BAB12269), Tj: *Tetraclita japonica formosana* (Lin et al., 2014, Biofouling, 30(2):169-181).
