# Supplementary material for: Molt-dependent transcriptomic analysis of cement proteins in the barnacle Amphibalanus amphitrite
Source: BMC Genomics. 2015 Oct 24;16:859. doi: 10.1186/s12864-015-2076-1 (PMC4619306; doi:10.1186/s12864-015-2076-1)
Supplement: Additional file 4: — RT-PCR primer sequences of 11 transcripts randomly selected from the differentially expressed transcripts and their gene functions. Primer sequences of the cement proteins and cytochrome b are also included. (PDF 17 kb) [file 12864_2015_2076_MOESM4_ESM.pdf]

Additional File 4. qRT-PCR primer sequences.

Primer ID	Primer Sequence	Gene function
**Molting protein**		
comp48323_c0_F	ATACAGCTGCTTCGCCTCAG	
comp48323_c0_R	CAAGACGCAAAGAGGCCCTA	Biogenesis of lysosome-related organelles complex 1 subunit 1
comp56413_c0_F	GACACCACCATCCTGTTCGT	
comp56413_c0_R	AGTCGTCCGATCCCTTGTTG	Translocon-associated protein subunit alpha
comp26034_c1_F	CTGATAGCCACCCTCGTTGG	
comp26034_c1_R	GAGCTGGACAACGGACAGAA	Larval cuticle protein LCP-14
comp43363_c1_F	TCGTCCAACAGCTCGGACTC	
comp43363_c1_R	CCTCCTCCTCCTCAGACACA	Sex comb on midleg-like protein 2
comp47010_c0_F	TGAGAGGTGATTGTGTGCCG	
comp47010_c0_R	ACCGTCTTCTATCCGTCCCA	unknown
comp44396_c2_F	TGAAAGGGTAGAGAGGCGGA	
comp44396_c2_R	AATGCGCCTAGATCACGTCC	Guanine nucleotide-binding protein subunit gamma
comp50890_c0_F	CTGGAGTCGGCGTAATACCC	
comp50890_c0_R	GAACCCCTCGGACTGTCATC	unknown
comp46667_c11_F	CCACACCCGGATCACTGAAA	
comp46667_c11_R	GAGTAGCCGCGTAGTCTGTC	
comp40054_c0_F	CCGAGTACACTGCCTGGATG	
comp40054_c0_R	ACTTCGAGCCGCAGTTCTAC	Apolipoprotein D
comp43913_c2_F	ATGGCAGCGTTCGAGTACAT	
comp43913_c2_R	ATCTTTGTCGGTGGCCTGAC	Tetraspanin-9
comp47469_c0_F	GCTGCGCATCACTTCTTTGA	
comp47469_c0_R	CTCTCAATGACGCCCCAGAG	unknown
Cement protein		
comp46913_c0_F	TTCAGCTCAAGGTTTTGCGC	Aacp114k
comp46913_c0_R	TCCAGGCTCAGGATGCTAGT	
comp40622_c0_F	GCTGAACGTGCTGCTCAAAA	Aacp100k
comp40622_c0_R	CACAATCAGCGACTCCTGGT	
comp47169_c0_F	CAAGGAGAAGAAGCAGGGCA	Aacp19k
comp47169_c0_R	CCCTTCAGCTTGAGTTCGGT	
comp26233_c0_F	GAACTGCAACCCAGGCAATC	Aacp20k-1
comp26233_c0_R	TTGCTCACGTGGATGTGGAA	
comp46760_c0_F	ACATGATCACGGCCACAAGT	Aacp20k-2
comp46760_c0_F	GGTTTGCACTGGTCATGCAG	
comp55903_c0_F	ATTCGGCAGCTGGTTCTGAA	Aacp52k
comp55903_c0_R	GGCGACGATGAATTTCACGG	
comp46764_c0_F	AGCTGTTTGGCTGACTGGAA	Cytochrome b (reference)
comp46764_c0_R	TGCCTAAAACTCAGCCACCT	
