# Supplementary material for: Molt-dependent transcriptomic analysis of cement proteins in the barnacle Amphibalanus amphitrite
Source: BMC Genomics. 2015 Oct 24;16:859. doi: 10.1186/s12864-015-2076-1 (PMC4619306; doi:10.1186/s12864-015-2076-1)
Supplement: Additional file 5: — ClustalX alignment of deduced cuticle protein sequences which encoding genes were highly expressed in the pre-molt stage. Rebers–Riddiford (RR) consensus sequence is highlighted. (PDF 183 kb) [file 12864_2015_2076_MOESM5_ESM.pdf]

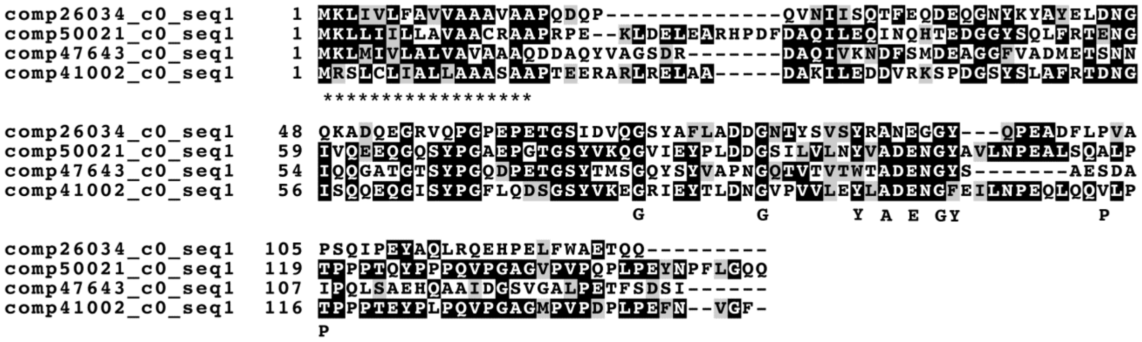

Additional File 5. ClustalX alignment of deduced cuticle protein sequences which encoding genes were highly expressed in the pre-molt stage. Rebers–Riddiford (RR) consensus sequence ($Gx_8Gx_7YxAxExGYx_7Px_2P$) is shown under the alignment. Star line (***…) represents signal peptide region.
