# Supplementary material for: Molt-dependent transcriptomic analysis of cement proteins in the barnacle Amphibalanus amphitrite
Source: BMC Genomics. 2015 Oct 24;16:859. doi: 10.1186/s12864-015-2076-1 (PMC4619306; doi:10.1186/s12864-015-2076-1)
Supplement: Additional file 8: — Comparison of sequence assemblies in the pre- and post-molt samples. (PDF 6 kb) [file 12864_2015_2076_MOESM8_ESM.pdf]

**Additional File 8.** Comparison of sequence assemblies in the pre- and post-molt samples.

| | Pre-molt | Post-molt | Combined[a] |
|---|---|---|---|
| Assembled contigs | 73,973 | 70,840 | 144,813 |
| Assembled sequence size (bp) | 41,874,597 | 43,469,940 | 85,344,537 |
| Clustered contigs (95%) | 64,025 | 57,871 | 95,873 |
| Clustered sequence size (bp) | 35,122,770 | 34,660,946 | 54,821,270 |

[a] Combination of the pre- and post-molt samples; CD-HIT clustering of assemblies with 95% sequence homology.
