# Supplementary material for: Molt-dependent transcriptomic analysis of cement proteins in the barnacle Amphibalanus amphitrite
Source: BMC Genomics. 2015 Oct 24;16:859. doi: 10.1186/s12864-015-2076-1 (PMC4619306; doi:10.1186/s12864-015-2076-1)
Supplement: Additional file 9: — Schematics and experimental conditions of the barnacle secretion collection methods. (PDF 537 kb) [file 12864_2015_2076_MOESM9_ESM.pdf]

Additional File 9

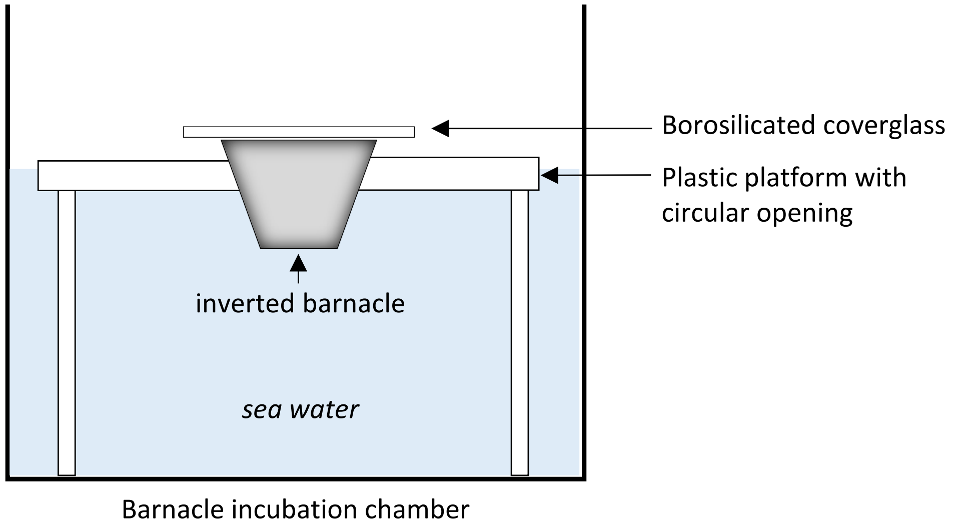

Coverslip samples. Schematic representation of setup used to collect barnacle cement on a glass coverslip for subsequent mass spectrometry analysis.

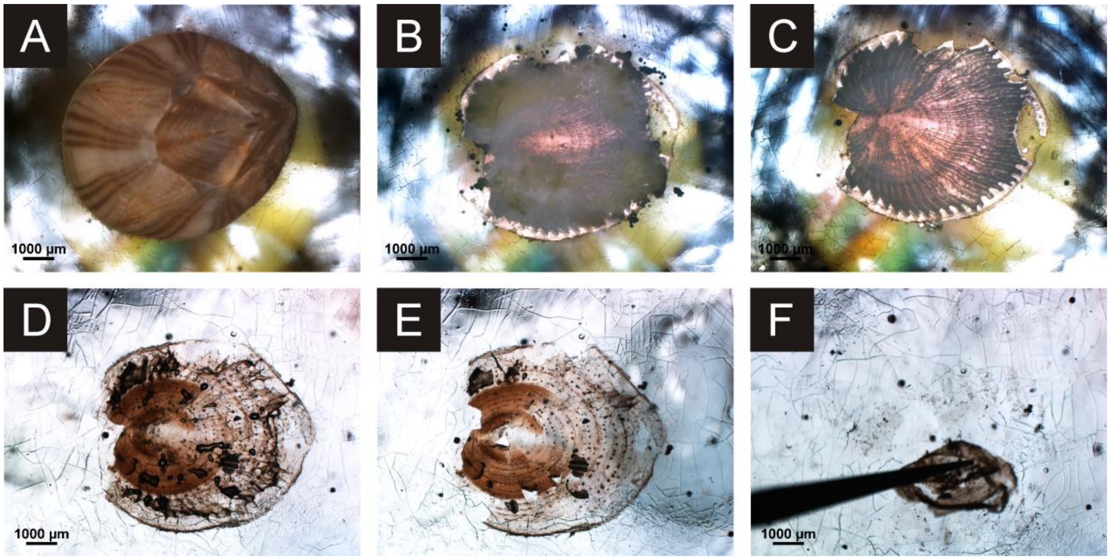

Motar and medallion samples. A) Optical microscopy image of a barnacle settled on a sodium aluminoborate glass substrate. B) Barnacle body and side walls were removed; sub-mantle tissue remained on top of baseplate. C) Mineralized barnacle base after the removal of sub-mantle tissue. D) Barnacle base after the baseplate was de-mineralized in a 0.1M EDTA solution; organic component of the baseplate, “mortar”, remained on top of the cuticular layer. E) Remaining interfacial layer, cuticle plus secreted layer, after the removal of the “mortar”. F) Interfacial layer, *i.e.* “medallion,” was peeled off the surface and processed for MS analysis.

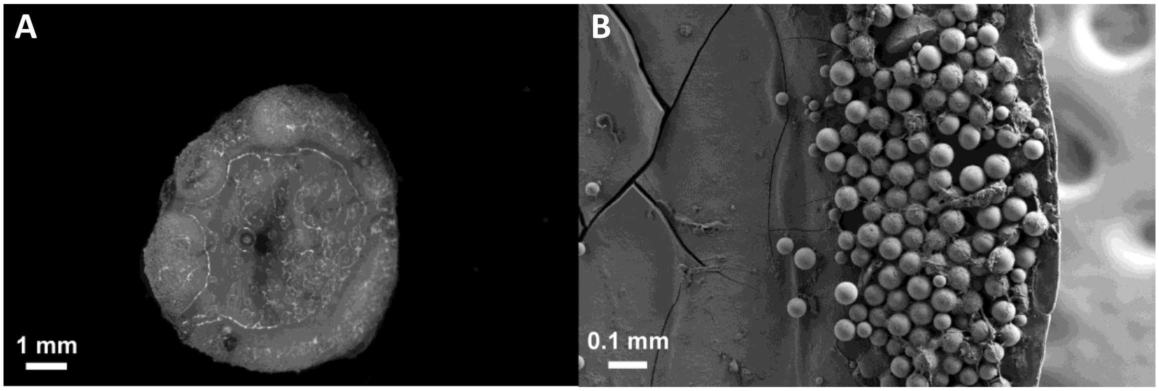

Bead samples. A) Optical microscopy image of the underside of a barnacle settled on a packed bed of glass microspheres for 24 hours. Barnacle secretions lead to the agglomeration of microspheres to the underside of the barnacle. B) Scanning electron microscopy image of the underside of the barnacle after microspheres were gently scraped off the bottom of the barnacle. Due to the adhesiveness of the barnacle secretions, microspheres remained adhered to the leading edge of the barnacle after scraping, and the barnacle was undamaged by the scraping.
